# GRP78在肺腺癌细胞顺铂耐药中的作用

**DOI:** 10.3779/j.issn.1009-3419.2010.01.07

**Published:** 2010-01-20

**Authors:** 晶 吴, 琪 王, 佳瑞 王, 黎川 张, 龙 赵

**Affiliations:** 1 116031 大连，大连市第四人民医院呼吸内科 Department of Respiratory Medicine, the Dalian No.4 People Hospital, Dalian 116031, China; 2 116031 大连，大连医科大学附属二院呼吸内科 Department of Respiratory Medicine, the Second Hospital of Dalian Medical University, Dalian 116031, China

**Keywords:** 葡萄糖调节蛋白78（GRP78）, 肺肿瘤, 顺铂, 耐药性, GRP78, Lung neoplasms, Cisplatin, Drug resistance

## Abstract

**背景与目的:**

糖调节蛋白78（GRP78）在肺癌细胞中表达增高并与其对化疗药物VP-16的耐药性有关，而GRP78的表达与其对化疗药物顺铂的耐药性是否相关，研究较少。本研究旨在通过检测GRP78在人肺腺癌细胞SPCA-1中的表达及顺铂作用后的细胞生存率，研究GRP78在肺腺癌细胞顺铂耐药中的作用。

**方法:**

依据是否应用诱导剂A23187将细胞分为实验组和对照组，采用RT-PCR、Western blot方法检测各组细胞中GRP78在核酸和蛋白水平的表达，利用MTT法测定细胞在顺铂作用下的生存率，分析GRP78在SPCA-1细胞顺铂耐药中的作用。

**结果:**

A23187可以明显诱导SPCA-1细胞中GRP78在核酸和蛋白水平的表达，且其表达水平与A23187具有一定的浓度依赖性，MTT检测显示：实验组细胞生存率明显低于对照组，亦呈一定的A23187浓度依赖性。

**结论:**

在人肺腺癌细胞SPCA-1中，诱导剂A23187可以诱导GRP78核酸和蛋白水平的表达，其表达与肺癌细胞在顺铂作用下的生存率呈负相关，表明GRP78可提高SPCA-1细胞对顺铂的敏感性，与肺癌细胞化疗耐药有一定的相关性。

## Abstract (en)

**Background and objective:**

The expression levels of glucose-regulated protein 78 (GRP78) were elevated and correlated with resistance to chemotherapy drug VP-16 in lung cancer cells. However, little is known about the relationship between its expression and resistance to cisplatin in lung cancer cells. The aim of this study was to investigate the expression of GRP78 under the induction of A23187 and its significance in the resistance to anti-tumor drugs cisplatin in a human lung cancer SPCA-1 cell line.

**Methods:**

RT-PCR and Western blot were used to analyze the expression of GRP78 at mRNA and protein levels in SPCA-1 cell line induced by A23187 at different concentrations (0, 1, 2, 4, 6 μM). MTT was used to determine the effect of cisplation on cell survival.

**Results:**

The expressions of GRP78 at both mRNA and protein levels were increased obviously in SPCA-1 cell line induced by A23187, with a manner of dose-dependent of A23187 to a great degree; MTT assay showed that the cell survival rate of the A23187-induced group decreased significantly compare to the control group, also with a concentrationdependent manner of A23187.

**Conclusion:**

The expression of GRP78 at both mRNA and protein levels were increased obviously in SPCA-1 cell line induced by A23187. The enhancement of GRP78 showed a negative correlation with the cell survival rate treated by cisplatin. All these indicated that overexpression of GRP78 can enhance the sensitivity to cisplatin and there is correlation between the expression of GRP78 and resistance to cisplatin of human lung cancer SPCA-1 cell line.

肺癌是最常见的恶性肿瘤之一，其发病率呈逐年升高趋势，而且80%的患者确诊时已丧失手术机会，因此化疗在肺癌的治疗中具有重要的作用。然而化疗耐药一直是肺癌治疗领域的热点问题，肿瘤耐药的发生与肿瘤细胞生存的微环境密切相关。由于肿瘤生长迅速，血液供应相对不足，产生了低糖、缺氧及酸中毒的微环境，这种微环境能够诱导糖调节蛋白78（glucose regulated protein, GRP78）的表达，而GRP78具有维持内质网稳定、保护细胞的作用^[1, 2]^。钙离子拮抗剂A23187是一种Ca^2+^转运载体，可以破坏细胞内质网内钙离子平衡，能够诱导肿瘤细胞合成GRP78^[3]^。有研究^[4]^表明GRP78在肿瘤细胞中呈现高表达，并与肿瘤细胞对化疗药物的耐药性有关。我们曾对大细胞肺癌、小细胞肺癌中GRP78的表达与VP-16耐药之间的相关性进行了研究^[5, 6]^。但关于GRP78在肺腺癌中的表达情况及其表达是否与腺癌细胞对化疗药物顺铂的耐药具有相关性，目前国内外研究较少。本研究旨在通过对在诱导剂A23187作用下肺腺癌细胞SPCA-1中GRP78表达水平的检测及其表达与肺癌细胞对顺铂耐药的相关性的分析，探讨GRP78在肺腺癌对顺铂耐药中的作用。

## 1 材料与方法

### 1.1 材料

#### 1.1.1 细胞系

人肺腺癌SPCA-1标准细胞株，购于中科院细胞研究所。

#### 1.1.2 试剂

RPMI-1640培养液、A23187、顺铂、Trizol（Sigma产品），RT-PCR试剂盒、DNA Marker DL2000（Takara产品），羊抗人GRP78抗体、羊抗人β-actin抗体、HRP-兔抗羊IgG（Santa Cruz公司），ECL试剂盒（Amershan公司），MTT（Amresco公司），DMSO、A23187、顺铂（Sigma公司）。

### 1.2 实验方法

#### 1.2.1 细胞培养及分组

SPCA-1细胞株复苏后培养于RPMI-1640培养基中，置于5%CO_2_、饱和湿度37 ℃的孵箱中培养。将5×10^4^个细胞接种于25 mL的培养瓶中培养24 h后，再将细胞分为实验组与对照组，实验组加入不同浓度A23187（1 μM、2 μM、4 μM、6 μM）；对照组加入PBS，培养24 h。

#### 1.2.2 RNA提取以及RT-PCR

按试剂盒说明分别提取实验组和对照组细胞的总RNA，制备cDNA，进行PCR产物扩增。GRP78 mRNA上游引物序列为5’GATAATCAACCAACTGTTAC 3’，下游引物序列为5’GTATCCTCTTCACCAGTTGG 3’，预计扩增片段长度为577bp；内参β-actin上游引物序列为5’TCGTCACCAACTGGGACGACATGG 3’，下游引物序列为5’GATCTTGATCTTCATTGTGCTGGG 3’，预计扩增片段长度为750 bp。PCR循环参数：94 ℃预变性4 min，94 ℃变性1 min，58 ℃退火30 s，72 ℃延伸30 s，30个循环，72 ℃再延伸10 min。PCR反应体系为：cDNA 2 μL、PCR引物各1 μL、5×PCR Buffer 10 μL、25 mmol/L MgCl_2_ 2 μL、dNTP混合物1 μL、Taq酶0.25 μL。取PCR扩增产物10 μL，于1.5%琼脂糖凝胶电泳，EB染色，用美国UVP凝胶成像系统EC3 System进行扫描分析，计算公式为：某样品GRP78 mRNA的相对表达水平=样品GRP78的IOD值/样品β-actin的IOD值。

#### 1.2.3 蛋白提取及Western杂交

将细胞置于1. 5 mL Eppendorf管中，加入RIPA缓冲液300 μL，以12 000 rpm速度、4 ℃离心10 min，将上清转移至新管，再重复离心2次，-70 ℃保存；Kobayashi等^[7]^使用Bradford比色法测定蛋白含量，依次进行SDS-PAGE、电转印。取下转印好的PVDF膜，放入封闭液中4 ℃封闭过夜，加入稀释的羊抗人GRP78抗体（一抗，1:400）及羊抗人β-actin（一抗，1:400），37 ℃孵育1 h，PBST洗膜3次，每次5 min。再加入稀释的辣根过氧化物酶标记兔抗羊IgG（二抗，1:5 000），37 oC孵育1 h，PBST洗膜3次，每次5 min，在室温进行ECL显色。用美国UVP公司EC3 System凝胶成像系统对PVDF膜进行扫描分析，计算公式为：某样品GRP78蛋白的相对表达水平=样品GRP78的IOD值/样品β-actin的IOD值。

#### 1.2.4 MTT比色法

取对数生长期的实验组和对照组SPCA-1细胞，用RPIM-1640培养基制成单细胞悬液，按1×10^4^个/孔接种于96孔培养板，每孔加200 μL培养基，置于5 %CO_2_、饱和湿度的37 ℃孵箱培养24 h。然后，加入不同浓度的顺铂（0 μM、2 μM、4 μM、6 μM、8 μM），每个浓度设1个主孔2个副孔，继续培养48 h后，再向各孔加入MTT 20 μL，4 h后加DMSO溶解甲臢，在酶标仪上选择波长490 nm，检测各孔OD值，计算细胞生存率及IC_50_。细胞生存率=（实验组OD值-空白对照组OD值）/（对照组OD值-空白对照组OD值）×100%；IC_50_：使用Bliss法计算；耐药指数（resistance index, RI）=IC_50_实验组/IC_50_对照组。

### 1.3 统计学处理

所有实验均重复3次以上，实验数据用MEAN±SD表示，采用SPSS 12.0软件处理，用单向方差分析比较多组间均数的差异，*P* < 0.05为差异有统计学意义。

## 2 结果

### 2.1 肺腺癌SPCA-1细胞中GRP78核酸水平的表达

RT-PCR结果显示：对照组GRP78核酸的相对表达量为0.165±0.0073；实验组中，当A23187浓度为1 μM-6 μM时，GRP78核酸的相对表达量分别为0.495±0.030、0.604±0.031、0.705±0.046和0.890±0.046；与对照组相比，其表达水平明显增高（*P* < 0.05），并呈现一定的A23187浓度依赖性（图 1）。

**1:**
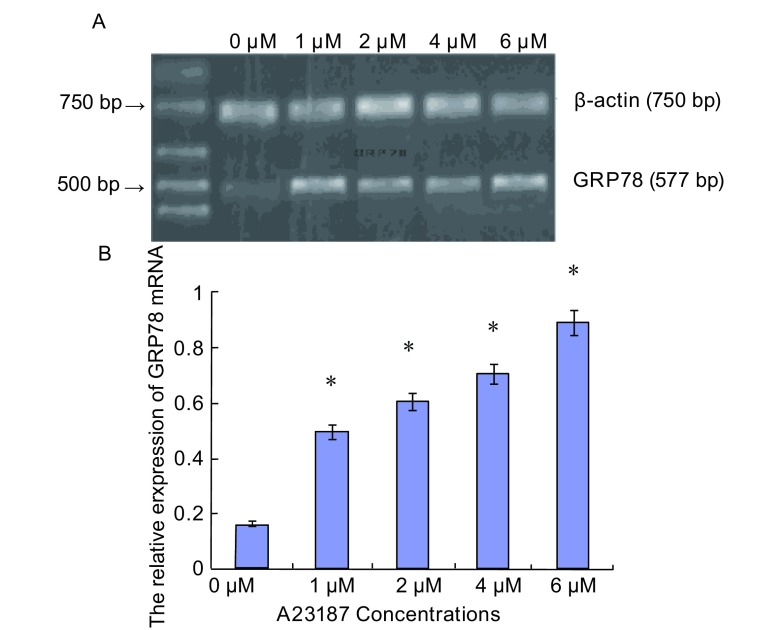
不同浓度A23187诱导下SPCA-1细胞GRP78核酸水平的表达 Expression of GRP78 mRNA in SPCA-1 cells exposed to A23187 at different concentrations

### 2.2 肺腺癌SPCA-1细胞中GRP78蛋白水平的表达

Western杂交结果显示：对照组GRP78蛋白的相对表达量为0.304±0.003；实验组中，当A23187浓度为1 μM-6 μM时，GRP78蛋白的相对表达量分别为0.545±0.004、0.763±0.012、0.864±0.023和0.892±0.034。与对照组相比，其表达水平明显增高（*P* < 0.05），并呈现一定的A23187浓度依赖性（图 2）。

**2:**
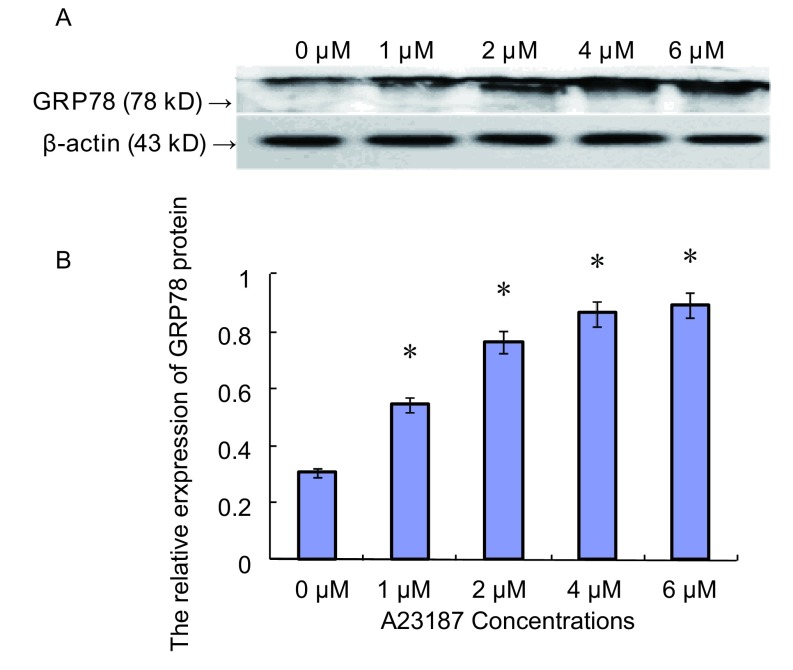
不同浓度A23187诱导下SPCA-1细胞GRP78蛋白水平的表达 Expression of GRP78 protein in SPCA-1 cells exposed to A23187 at different concentrations

### 2.3 细胞生存率与GRP78表达的相关性

不同浓度A23187诱导下细胞对顺铂的IC_50_值。对照组IC_50_为5.73±0.43；实验组中，当A23187浓度为1 μM、2 μM、4 μM、6 μM时，其IC_50_值分别为：7.06±0.37、4.23±0.17、3.84±0.32和3.03±0.24。可见在相同浓度顺铂作用下，当A23187浓度为2 μM、4 μM、6 μM时，其所对应的细胞生存率明显低于对照组（*P* < 0.05），且其降低呈现一定的A23187浓度依赖性。结合前述GRP78在核酸和蛋白水平表达的特点，即A23187对GRP78的表达呈浓度依赖性诱导，提示肺腺癌细胞对顺铂的耐药性与GRP78表达呈负相关（图 3）。

**3:**
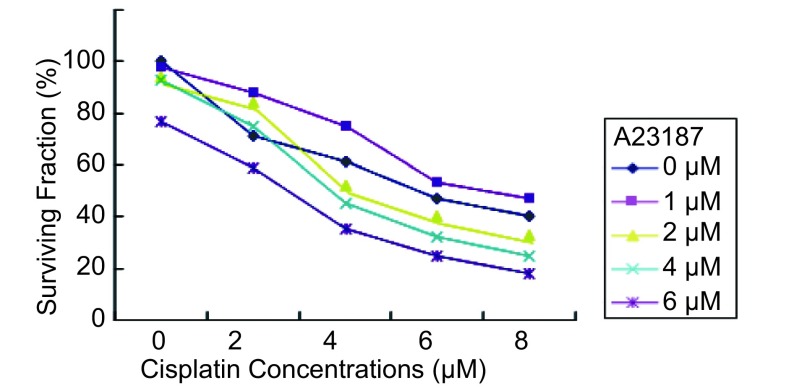
不同浓度A23187诱导下的SPCA-1细胞对顺铂的生存曲线 Survival curves of SPCA-1 cells to cisplatin after being induced by A23187 at different concentrations

## 3 讨论

GRP78具有促进错误折叠蛋白恢复正常构象、维持内质网和胞质Ca^2+^平衡并调节内质网内Ca^2+^依赖性蛋白修饰反应、对抗氧化应激，从而保护细胞的作用^[8]^。各种应激条件如葡萄糖缺乏、蛋白错误折叠、蛋白糖基化及一些诱导剂（如Ca^2+^-ATPase抑制剂thapsigargin）都可以激活GRP78转录基因，诱导产生GRP78^[3, 9, 10]^。近年来的研究^[11]^发现应激条件下细胞合成和高度表达GRP78的反应，可能是细胞的一种重要防御机制。该机制对细胞具有保护作用，能够延长在各种不利因素刺激下的细胞生存期^[12-14]^。而且，GRP78在一些肿瘤细胞中呈高表达，对肿瘤细胞的耐药性有一定影响。

A23187是一种Ca^2+^转运载体，作用于细胞后，可使细胞内质网Ca^2^+浓度（[Ca^2+^]ER）下降，胞质内Ca^2+^浓度（[Ca^2+^]c）增加。因而，可以造成内质网Ca^2+^失衡，产生内质网应激反应，激活*GRP78*基因，诱导产生GRP78^[3]^。已有研究发现A23187可以诱导人大细胞肺癌细胞NCI-H460及人小细胞肺癌细胞NCI-H446 GRP78的表达^[5, 6]^。本研究表明：A23187也可以明显诱导人肺腺癌SPCA-1细胞GRP78在蛋白及核酸水平的表达。

顺铂属细胞周期非特异性药物，其抗癌谱广，自70年代开始用于临床以来，已成为治疗肺癌、食管癌、恶性淋巴瘤、卵巢癌等多种恶性肿瘤的一线化疗药物。本实验的结果显示，当A23187浓度为2 μM、4 μM、6 μM时，SPCA-1细胞中GRP78的表达明显升高，相应的细胞在顺铂作用下的生存率却明显降低，表明A23187诱导下的GRP78高表达与SPCA-1细胞顺铂耐药呈现负相关性，即GRP78高表达能够提高肺腺癌细胞对顺铂的敏感性。Mese等^[15]^通过对人表皮细胞癌的研究发现，GRP78表达增高能够提高野生株A431细胞对顺铂的敏感性，而对耐药株A431/CDDP2细胞没有影响。Belfi等^[16]^利用实验证明人结肠癌细胞HCT116、SW480与VACO-8中GRP78的表达增高，可以增强结肠癌细胞对顺铂的敏感性。Satadal等^[17]^通过对中国仓鼠V79细胞的研究发现，在两种诱导剂6-氨基烟碱与2-脱氧葡萄糖的作用下，GRP78的表达水平均明显增高，与此相对应，V79细胞对顺铂的敏感性亦明显增强。顺铂主要通过与DNA分子形成链内或链间交叉联接或阻止RNA分子再复制等途径发挥抗肿瘤药理作用，GRP78高表达能够提高细胞对顺铂的敏感性的机制主要与DNA交联修复有关。一方面，GRP78能够减慢DNA交联产物从细胞内脱失的速度；另一方面，GRP78还能够削弱DNA加和物切补修复，从而导致DNA交联产物在细胞内滞留时间延迟，增强药物的敏感性^[17]^。

当然，肺癌细胞对顺铂的耐药是多因素多因子共同参与的复杂过程，除了细胞微环境中的GRP78的作用以外，其它机制如药物流入减少或流出增加、与谷胱甘肽或金属硫蛋白结合、机体对药物的解毒作用增强、DNA修复以及DNA复制损伤等也与此相关。因此，肺癌顺铂耐药机制值得进一步研究。

## References

[1] Gazit G, Hung G, Chen X (1999). Use of the glucose-starvation inducible grp78 promoter in suicide gene therapy of murine fibrosarcoma. Cancer Res.

[2] Cai JW, Henderson BW, Shen JW (1993). Induction of glucose regulated proteins during growth of a murine tumor. J Cell Physiol.

[3] Li WW, Alexandre S, Cao X (1993). Transactivation of the grp78 promoter by Ca^2+^ depletion: A comparative analysis with A23187 and the endoplasmic reticulum Ca(^2+^)-ATPase inhibitor thapsigargin. J Biol Chem.

[4] Lee AS (2001). The glucose-regulated proteins: stress induction and clinical applications. Trends Biochem Sci.

[5] Wang Q, Wang T, Wang Y (2007). VP-16 resistance in the NCI-H460 human lung cancer cell line is significantly associated with glucoseregulated protein78 (GRP78) induction. Anticancer Res.

[6] Wang Y, Wang W, Wang S (2008). Down-regulation of GRP78 is associated with the sensitivity of chemotherapy to VP-16 in small cell lung cancer NCI-H446 cells. BMC Cancer.

[7] Kobayashi H, Man S, Graham CH (1993). Acquired multicellular-mediated resistance to alkylating agents in cancer. Proc Natl Acad Sci USA.

[8] Buchner J (1996). Supervising the fold: functional principles of molecular chaperones. FASEB J.

[9] Wu KD, Bunqard D, Lytton J (2001). Regulation of SERCA Ca^2+^ pump expression by cytoplasmic Ca^2+^ in vascular smooth muscle cells. Am J Physiol Cell Physiol.

[10] Ciocca DR, Calderwood SK (2005). Heat shock proteins in cancer: diagnostic, prognostic, predictive, and treatment implications. Cell Stress Chaperones.

[11] Cai B, Tomida A, Mikami K (1998). Down-regulation of epidermal growth factor receptor-signaling pathway by binding of GRP78/Bip to the receptor under glucose-starved stress conditions. J Cell Physiol.

[12] Jacquier-Sarlin MR, Dreher D, Polla BS (1996). Selective induction of the glucose-regulated protein grp78 in human monocytes by bacterial extracts(OM-85): a role for calcium as second messenger. Biochem Biophys Res Commun.

[13] Jamora C, Dennert G, Lee AS (1996). Inhibition of tumor progression by suppression of stress protein GRP78/Bip induction in fibrosarcoma B/C10ME. Proc Natl Acad Sci USA.

[14] Yang GH, Li S, Pestka JJ (2000). Down-regulation of the endoplasmic reticulum chaperone GRP78/Bip by vomitoxin (Deoxynivalenol). Toxicol Appl Pharmacol.

[15] Mese H, Sasaki A, Nakayama S (2001). Analysis of cellular sensitization with cisplatin-induced apoptosis by glucose-starved stress in cisplatin-sensitive and -resistant A431 cell line. Anticancer Res.

[16] Belfi CA, Chatterjee S, Gosky DM (1999). Increased sensitivity of human colon cancer cells to DNA cross-linking agents after GRP78 upregulation. Biochem Biophys Res Commun.

[17] Satadal Chatterjee, Haruyo Hirota, Charles A (1997). Hypersensitivity to DNA cross-linking agents associated with up-regulation of glucose-regulated stress protein GRP78. Cancer Res.

